# Doxycycline or Ofloxacin for Outpatient Chlamydial Pelvic Inflammatory Disease? A Cost-Benefit and Cost-Effectiveness Analysis

**DOI:** 10.1155/S106474499500024X

**Published:** 1995

**Authors:** Michael J. Rosenberg, Michael S. Waugh

**Affiliations:** 1 Health Decisions, Inc. 100 Europa Drive Suite 525 Chapel Hill NC 27515 USA; 2 Departments of Epidemiology and Obstetrics-Gynecology University of North Carolina Chapel Hill NC USA

## Abstract

*Objective:* The current Centers for Disease Control and Prevention (CDC) guidelines include 2 drugs, doxycycline and ofloxacin, for treatment of the chlamydial component of outpatient pelvic inflammatory disease (PID). Although ofloxacin costs about $90 more than doxycycline, doxycycline is frequently associated with side effects and patient compliance with this drug is probably poor. Because clinicians have little information by which to judge the tradeoffs between price and compliance for these 2 antibiotics, we examined the impact of patient compliance in the evaluation of the costs and benefits of using each drug.

*Methods:* The incidence and direct costs of PID sequelae (infertility, ectopic pregnancy, and chronic pelvic pain) resulting after partially treated chlamydial PID were taken from previous estimates. For differing levels of antibiotic compliance, the probability of cure, probability of the occurrence of sequelae, and the associated cost of each were calculated. Because the relationship between partial antibiotic compliance and PID cure is unknown, we included 3 plausible relationships in our analyses. The sensitivity analysis was performed by varying key assumptions and examining the effect of each on future costs.

*Results:* The average probability of future PID sequelae attributable to chlamydia is slightly less than 2%, with an associated cost of $1,272. With an average compliance for doxycycline of 50%, an improvement in compliance of as little as 1.8–3.5 percentage points (51.8–53.5%), depending on the assumption used regarding partial compliance and cure, would make the use of ofloxacin less costly than doxycycline in the long run. Even with a cost difference of $90 between the 2 drugs, a 10-percentage-point increase in compliance (to 60% compliance) with the more expensive drug would save $2.63 for each $1.00 spent.

*Conclusions:* Since the long-term costs of PID are likely to overshadow the immediate cost of providing treatment, physicians should carefully consider the likelihood of patient compliance in selecting an antibiotic.


					Infectious Diseases in Obstetrics and Gynecology 3:12-21 (1995)
(C) 1995 Wiley-Liss, Inc.
Doxycycline or Ofloxacin for Outpatient Chlamydial
Pelvic Inflammatory Disease?
A Cost-Benefit and Cost-Effectiveness Analysis
Michael J. Rosenberg and Michael S. Waugh
Health Decisions, Inc. (M.J.R., M.S.W.), and Departments ofEpidemiology and
Obstetrics-Gynecology, University ofNorth Carolina (M.J.R.), Chapel Hill, NC
ABSTRACT
Objective: The current Centers for Disease Control and Prevention (CDC) guidelines include 2
drugs, doxycycline and ofloxacin, for treatment of the chlamydial component of outpatient pelvic
inflammatory disease (PID). Although ofloxacin costs about $90 more than doxycycline, doxycy-
cline is frequently associated with side effects and patient compliance with this drug is probably poor.
Because clinicians have little information by which to judge the tradeoffs between price and compli-
ance for these 2 antibiotics, we examined the impact of patient compliance in the evaluation of the
costs and benefits ofusing each drug.
Methods: The incidence and direct costs of PID sequelae (infertility, ectopic pregnancy, and
chronic pelvic pain) resulting after partially treated chlamydial PID were taken from previous
estimates. For differing levels of antibiotic compliance, the probability of cure, probability of the
occurrence of sequelae, and the associated cost of each were calculated. Because the relationship
between partial antibiotic compliance and PID cure is unknown, we included 3 plausible relation-
ships in our analyses. The sensitivity analysis was performed by varying key assumptions and
examining the effect of each on future costs.
Results: The average probability of future PID sequelae attributable to chlamydia is slightly less
than 2%, with an associated cost of $1,272. With an average compliance for doxycycline of 50%, an
improvement in compliance of as little as 1.8-3.5 percentage points (51.8-53.5%), depending on the
assumption used regarding partial compliance and cure, would make the use of ofloxacin less costly
than doxycycline in the long run. Even with a cost difference of $90 between the 2 drugs, a
10-percentage-point increase in compliance (to 60% compliance) with the more expensive drug
would save $2.63 for each $1.00 spent.
Conclusions: Since the long-term costs of PID are likely to overshadow the immediate cost of
providing treatment, physicians should carefully consider the likelihood of patient compliance in
selecting an antibiotic. (C) 1995 Wiley-Liss, Inc.
KEY WORDS
Antibiotic compliance, infertility, ectopic pregnancy, chronic pelvic pain
elvic inflammatory disease (PID) is a serious
reproductive health problem in the United
States. Each year, an estimated 1.28 million women
are treated as outpatients for episodes ofacute PID.
Direct and indirect costs, the latter including lost
wages and lost value ofhousehold management, are
estimated at $1.3 billion annually for these cases.
In addition to these costs, PID produces sequelae
Address correspondence/reprint requests to Dr. Michael J. Rosenberg, Health Decisions, Inc., 100 Europa Drive, Suite 525,
Chapel Hill, NC 27515.
Portions of this work were presented at the Interscience Conference on Antimicrobials and Chemotherapy, Orlando, FL,
October 5, 1994.
Received November 10, 1994
Clinical Study Accepted May 12, 1995
DOXYCYCLINE OR OFLOXACIN FOR OUTPATIENT PID? ROSENBERG AND WAUGH
that are often delayed, in the form of chronic pelvic
pain, infertility, and ectopic pregnancy; these com-
ponents add an additional annual cost of $3.8 bil-
lion.
Chlamydia trachomatis is an important contribu-
tor to PID. In the United States, it has been identi-
fied in 38% of hospitalized cases2
and in as many as
52% of women treated as outpatients.3'4 Infection
with C. trachomatis is perhaps more insidious than
with other forms of PID because of its less symp-
tomatic nature. For example, Svennson et al.s
found
that patients with chlamydial salpingitis often pre-
sented after a longer period (7-9 days) of abdomi-
nal pain. These patients less often had fever, but
more often had elevated sedimentation rates (often
to 30-50 mm/h). Yet, while the clinical findings
were not impressive in patients with chlamydial
salpingitis, laparoscopy revealed more pronounced
inflammation of the fallopian tubes than expected
from the clinical picture. Similarly, a higher pro-
portion of women with chlamydial cervical infec-
tion had more demonstrable tubal infection than
women with gonorrheal cervical infection.6
Because infection can rapidly cause irreversible
tubal damage, antibiotics must be immediately
started when PID is suspected. Many physicians
intentionally overtreat, recognizing the difficulty
of identifying an underlying salpingitis or endo-
metritis based on physical and laboratory findings.7
Since few women diagnosed with PID, especially
those treated as outpatients, undergo laparoscopy or
other diagnostic or laboratory procedures that might
help identify the microbiologic etiology, the treat-
ment is by necessity empiric and broad enough to
cover C. trachomatis as well as other pathogens
linked etiologically with PID. Currently, the Cen-
ters for Disease Control and Prevention (CDC)
recommends 2 regimens that include either doxycy-
cline or ofloxacin for coverage of C. trachomatis.
Both are highly effective against C. trachomatis,
but ofloxacin costs approximately $90 more than
doxycycline. 8
The effectiveness of either drug depends on how
it is used. Patient compliance depends on a number
of factors, including the occurrence of side effects,
the degree of symptomaticity (perceived need), and
the individual's degree of conscientiousness and
health awareness. Even for medications that are
regularly taken, compliance may be considerably
less than optimal. For example, approximately one-
fourth of patients using medications to prevent sei-
zures, for whom the consequences of noncompli-
ance are severe, did not comply with their
prescribed regimen.9
Given the minimal degree of
symptoms often associated with outpatient PID,
particularly after the onset of symptom relief, the
patient's perceived need to continue treatment may
be reduced.
The few studies ofdrug compliance for the treat-
ment of sexually transmitted diseases (STDs) sup-
port the notion that patient compliance is poor. One
study ofPID treatment in patients seen in an urban
emergency department indicated an average com-
pliance of only 50% with the doxycycline regimen
(b.i.d. for 7 days). The occurrence of side effects
was the strongest factor in compliance. 10
A second
study of erythromycin treatment for chlamydia and
gonorrhea likewise indicated that the occurrence of
side effects increased noncompliance. 11
For acute infections, the occurrence of side ef-
fects may be a key determinant of compliance, and
the limited evidence available suggests that doxycy-
cline and ofloxacin differ in this regard. Two stud-
ies found that side effects were more prevalent with
doxycycline regimens than with ofloxacin regi-
mens. lz'13 In these 2 comparative trials of oral
ofloxacin (400 mg b.i.d, for 10 days) or doxycy-
cline (100 mg b.i.d, for 10 days), the side effects
were approximately twice as common in the doxy-
cycline group than in the ofloxacin group (respec-
tively, 15% vs. 7% in one study and 26% vs. 16%
in the other). Nausea and other gastrointestinal dis-
turbances are commonly recognized as side effects
ofdoxycycline and other tetracyclines. In the larger
of the 2 studies, nausea and vomiting were signifi-
cantly more common among patients using the dox-
ycycline regimen, lz
A clinician treating outpatient PID has little
information on which to weigh the tradeoffs be-
tween patient compliance and cost. To address this
issue, we present an analysis of the effect of patient
compliance with doxycycline and ofloxacin on the
future occurrence and cost of sequelae resulting
from chlamydial PID.
MATERIALS AND METHODS
Costs of Sequelae
The estimates of the costs of PID sequelae (chronic
pelvic pain, ectopic pregnancy, and infertility) were
taken from Washington and Katz. The direct costs
INFECTIOUS DISEASES IN OBSTETRICS AND GYNECOLOGY
DOXYCYCLINE OR OFLOXACIN FOR OUTPATIENT PID? ROSENBERG AND WAUGH
per patient, reported in 1990, were inflated to 1995
dollars using the medical care component of the
consumer price index by the formula "19955
1990$ 1.081 n,,, where n equals the elapsed num-
ber of years. These costs were then discounted to
account for events occurring in the future by the
formula "present value (19955 1.081 n)/
(1.04)n, where n represents the estimated number
ofyears in the future when the event will occur and
4% is the average rate of inflation for all items
reported in the consumer price index. Chronic pel-
vic pain, including acute events such as sal'pingitis
and tubo-ovarian abscess, is assumed to occur 3
years after the patient presents with PID; ectopic
pregnancy is assumed to occur in 5 years; and infer-
tility in 10 years. Thus, the present cost per case for
each condition was $15,962 for ectopic pregnancy,
$2,010 for infertility, and $14,754 for chronic
pelvic pain. Indirect costs, which refer to lost pro-
ductivity and represent the value of output forgone
by women with PID sequelae, were not considered
in this analysis.
Incidence of PID and Its Sequelae
The estimates of the incidence of PID and PID
sequelae were taken from the Hospital Discharge
Survey conducted by the National Center for Health
Statistics14
and from Washington and Katz. The
latter rely on data published by the National Dis-
ease Therapeutic Index, which includes only initial
visits to office-based physicians by women aged
15-44 years. Although the number of outpatient
PID cases reported in 1993 by office-based physi-
cians has declined by 7% since 1988, this decline
has been offset by an increase in the number of
women relying on health maintenance organiza-
tions (HMOs), clinics, and emergency depart-
ments, leaving the overall incidence unchanged (T.
MacKay, CDC, personal communications).
The number of women hospitalized for PID in
1991 was used to estimate the total number of cases
ofchronic pelvic pain. 14
The estimate ofthe annual
incidence ofPID-related ectopic pregnancy in 1991
was based on an assumption that 50% of the mor-
bidity and mortality of ectopic pregnancy reported
in the Hospital Discharge Survey is caused by
PID. Along with the results of a study of women
with laparoscopically verified PID who were fol-
lowed for 10 years, we used Washington and Katz
as our source in estimating that 20% ofwomen with
PID will become infertile, with half(10%) ofthese
PID cases attributable to chlamydial PID. 1,15
Patient Compliance
By average compliance, we mean the proportion of
days, for all patients, that the medication was taken
as prescribed. We found only one study of compli-
ance with doxycycline for PID. This study, in an
urban emergency department, found an average
compliance of approximately 50%.1 Complete
compliance for the 7-day b.i.d, regimen was re-
ported by 31% of the 386 patients surveyed, while
28% reported that they did not have their prescrip-
tions for doxycycline filled. The remaining 41%
reported that they stopped their medication early,
after an average of 4.1 days. These side effects of
doxycycline were the major determinants of non-
compliance in patients who discontinued treatment
early. No data were available regarding patient
compliance with ofloxacin.
Because the relationship between partial antibi-
otic compliance and PID cure is unknown, we
examined 3 plausible, but hypothetical, relation-
ships (Fig. 1). For each relationship, we assumed
that the probability ofcure for chlamydia was equal
for both doxycycline and ofloxacin at any given
level of compliance. Full compliance was assumed
to result in slightly less than full cure because, in
some cases, even the early initiation of antibiotic
therapy will be too late to prevent future problems,
as reported in a number of studies. 12,13,16
The
clinical response in women with positive chlamy-
dial cultures who presented with PID was esti-
mated at 96%, given full compliance. This estimate
was based on an average of reported cure rates [18
of 1812 and 6 of 713 patients cured with ofloxacin
(96%) and 15 of" 1712 and 10 of 1013 cured with
doxycycline (93%)] when the regimens were taken
as prescribed. Given the small number of patients
and the absence of a significant difference in the
clinical response, we used the higher average of
reported cure rates.
Change in Frequency and Cost of PID Sequelae
With Compliance
For a given level of compliance and a given rela-
tionship between partial compliance and cure (Fig.
1), the frequency and cost of chronic pelvic pain,
ectopic pregnancy, and infertility were determined
as follows. First, the probability of noncure was
14 INFECTIOUS DISEASES IN OBSTETRICS AND GYNECOLOGY
DOXYCYCLINE OR OFLOXACIN FOR OUTPATIENT PID? ROSENBERG AND WAUGH
0
0.80
0.70
.0.50
0.40
0.30
0.20
0.10
Assumption C
,./X
/
/
/
/ ///
///
//"
Assumption B // AssumptionA
0o4 20% 30o4 40% 50o4 60o4 70% 80% 90*/,
Patient compliance
100%
Fig. I. Three possible relationships between patient compliance and cure. Reduction of sequelae by
increased drug compliance for each assumption calculated as discussed (see Materials and Methods).
determined from Figure 1. For example, under
assumption A, a 50% compliance is associated with
a 0.52 probability of noncure. Second, the number
of patients receiving a diagnosis of outpatient PID
who were not cured was determined by multiplying
the probability of noncure by the number of PID
diagnoses each year (0.52 1.277 million
660,400). Third, the probability of developing a
given outcome was calculated by dividing the aver-
age probability of having that outcome, given a
diagnosis of outpatient PID, by the probability of
noncure. For ectopic pregnancy, this was 0.017/
0.52 0.033 (3.3%). Fourth, the number ofcases
ofeach outcome, such as ectopic pregnancy, was the
product of the number of individuals who are not
cured and the probability of developing an ectopic
pregnancy among women who are not cured. For
ectopic pregnancy, this was 660,400 0.033
22,000. Finally, the total cost of each outcome
resulting from noncure due to inadequate compli-
ance was the number of cases times the total direct
cost per case. Again, for ectopic pregnancy, this
costwas 22,000 $15,962 $351 million. The
results ofthese calculations for each ofthe outcomes
(ectopic pregnancy, infertility, and chronic pelvic
pain), using one hypothetical relationship between
compliance and the probability ofcure (assumption
A, Fig. 1), are summarized in Table 1. Identical
calculations were performed for each hypothetical
relationship between compliance and the probabil-
ity of cure (assumptions B and C, Fig. 1, data not
shown); the results are summarized in Figures 2
and 3.
RESULTS
With 1.28 million women treated as outpatients
each year for PID1'14 and an estimated 50% of
these (half of all PID sequelae) primarily attribut-
able to C. trachomatis,3'4 the probabilities of hav-
ing an ectopic pregnancy, becoming infertile, or
suffering chronic pelvic pain from chlamydia-asso-
ciated PID, given a diagnosis of outpatient PID,
were estimated at 1.7%, 10%, and 5.4%, respec-
tively (Table 2)' Using the total direct costs per
case for each of the 3 PID sequelae, the average
costs (total direct cost per case multiplied by the
INFECTIOUS DISEASES IN OBSTETRICS AND GYNECOLOGY
DOXYCYCLINE OR OFLOXACIN FOR OUTPATIENT PID? ROSENBERG AND WAUGH
probability of developing the condition, given a
diagnosis of PID for outpatient treatment) were
$276, $201, and $795, respectively; the total for
all 3 diagnoses was $1,272.
The probability of developing future complica-
tions is, however, affected by antibiotic compli-
ance. Full compliance by all patients was presumed
to cure nearly all chlamydial infections, resulting in
an average cost of all 3 later complications of $86.
The small probability of later complications even
with full compliance reflects the fact that, in some
patients, damage will already have occurred even
though treatment is promptly initiated. Zero com-
pliance was presumed to cure none, with an average
cost of $2,458. Between these 2 extremes, different
levels of compliance were associated with different
numbers and costs of PID sequelae. Table 1, mod-
eled for a single compliance-cure relationship, sum-
marizes how the cost of each sequelae of inade-
quately treated PID depends on compliance. The
differences in compliance from the 50% baseline
substantially changed both the number of cases and
the cost. For example, an increase in compliance
from the 50% baseline to 60% for ectopic preg-
nancy would decrease the number ofcases by 4,121
and the cost by $65.8 million each year (Table 1).
Despite the greater initial expense of ofloxacin,
treatment with this drug would be cost-beneficial if
it could increase patient compliance by 3.5 percent-
age points or more over the 50% baseline compli-
ance estimate for doxycycline (Fig. 2). Again, de-
pending on which relationship between partial
compliance and cure is analyzed, this increase might
be as low as 1.8 percentage points for ofloxacin to
be cost-beneficial. Using the most conservative of
the 3 relationships tested (assumption A), an in-
crease in average compliance from 50% to 60% (10
percentage points) results in an average cost savings
of $237 which, offset by an increase in expendi-
tures of $90 per patient, yields a savings-to-cost
ratio of almost $2.63 for each $1.00 spent. Like-
wise, an increase of 10 percentage points in compli-
ance results in a reduction of 18.6% (41,000 cases)
in the prevalence ofsequelae (Fig. 3). Overall, this
hypothetical increase of 10 percentage points in
compliance could result in a savings of over $303
million each year through a reduction in failed
treatments and the prevention of PID sequelae, at
an annual cost of almost $115 million for the more
expensive ofloxacin.
INFECTIOUS DISEASES IN OBSTETRICS AND GYNECOLOGY
%
U
<8
DOXYCYCLINE OR OFLOXACIN FOR OUTPATIENT PID? ROSENBERG AND WAUGH
1200
II00
I000
900
8OO
700
600
5OO
4O0
300
20O
100
0
o% 5% 10% 15% 20% 25% 30% 35%
Percentage point increase in compliance over 50% baseline
40%
Fig. 2. Average savings per patient for PID sequelae by increased drug compliance. Reduction of
sequelae by increased drug compliance for each assumption calculated as discussed (see Materials and
Methods).
250,000
200,000
150,000
I00,000
Assumption
Assemplaon
50,000
0%
Assumption C
5% 10% 15% 20% 25%
Percentage point increase in compliance over 50% baseline
3O%
Fig. 3. Reduction in incidence of PID sequelae by increased drug compliance. Reduction ofsequelae
by increased drug compliance for each assumption calculated as discussed (see Materials and Meth-
ods).
Sensitivity Analysis
To determine how changing the key assumptions in
the analysis might affect the conclusions, we exam-
ined a range ofplausible values for each assumption
(Table 3). The 3 primary components of the analy-
sis were baseline compliance with doxycycline, the
frequency of PID sequelae, and the costs of these
sequela. Changes of +20% in each ofthese compo-
INFECTIOUS DISEASES 1N OBSTETRICS AND GYNECOLOGY 17
DOXYCYCLINE OR OFLOXACIN FOR OUTPATIENT PID? ROSENBERG AND WAUGH
TABLE 2. Average frequency and cost of PID sequelae per patient, given outpatient diagnosis of PID
Ectopic Chronic
pregnancy Infertility pelvic pain Total
No. of cases per year
No. of cases attributable to chlamydia
Probability of developing condition,
given outpatient diagnosis of PID
Cost per case
Average cost, given outpatient diagnosis
of PID
44,200 255,500 137,720 437,420
22, 100 127,750 68,860 218,710
0.017 0. 100 0.054
$15,962 $ 2,010 $ 14,754
$ 276 $ 201 $ 795 $ 1,272
aSee refs. land 14.
bAssuming 50% of all cases of PID are attributable to chlamydial infection. See refs. 3 and 4.
cCosts per case estimated from ref. I.
nents had minimal impact on the level of improved
compliance necessary with ofloxacin to make its use
cost-beneficial (the break-even point).
Changing the baseline compliance with doxycy-
cline had no impact on our findings if the relation-
ship between partial compliance and cure were lin-
ear (assumption A, Fig. 1). However, increasing
the baseline compliance level by 20 percentage
points increased the break-even point (assumptions
B and C, Fig. 1), from 1.8% to 3.5% and from
2.3% to 4.5%. Assuming the compliance with dox-
ycycline to be 20 percentage points less than we
assumed, we observed similar effects on the break-
even point. Varying other parameters, such as in-
creasing or decreasing the costs and frequency of
sequelae by 20 percentage points, similarly had
minimal effects. Increases in the cost or frequency
of sequelae reduced the level of compliance neces-
sary to make the prescription of ofloxacin cost-
beneficial by percentage point or less. Decreasing
the cost or frequency of sequelae had an opposite,
still negligible, effect.
DISCUSSION
Because inadequately treated PID due to C. tra-
chomatis leads to expensive problems, even slight
improvements in compliance would be associated
with substantial reductions in the incidence and cost
of later complications. In the choice between the 2
recommended drugs for treating the chlamydial
component ofPID, an improvement in compliance
of as little as 1.8-3.5 percentage points over the
50% baseline (to 51.8-53.5%) justifies the use of
ofloxacin, which costs $90 more than doxycycline.
Even with such a cost discrepancy, an increase of as
little as 10 percentage points in compliance with the
more expensive drug would save $2.63 for each
$1.00 spent. These estimates may be conservative
because they do not include the indirect costs associ-
ated with PID sequelae. The inclusion of indirect
costs would likely increase the cost savings associ-
ated with improved compliance. A sensitivity anal-
ysis confirmed these conclusions, regardless of how
we varied the assumptions.
The strong financial impact of even slight im-
provements in patient compliance emphasizes the
high costs of PID sequelae. Although the average
probability of developing chronic pelvic pain, ec-
topic pregnancy, or infertility in a patient with
outpatient PID is relatively low and not every pa-
tient who develops a problem will seek medical
treatment, our calculations consider both these fac-
tors and emphasize the fact that each problem ne-
cessitates time-consuming, expensive, often com-
plex procedures frequently over extended periods.
In addition, the emotional toll for patients may be
considerable. The cost of PID sequelae and the
importance of adequate antibiotic treatment can be
appreciated by the fact that the CDC recommends
hospitalization for adolescents and other groups in
17
whom compliance is traditionally poor.
Our analysis is based on several assumptions.
First, only minimal information is available about
compliance with doxycycline and no comparative
information is available for ofloxacin. Whether or
not ofloxacin is associated with a better compliance
rate than doxycycline does not alter the validity of
the analysis, which emphasizes the importance and
costliness of poor compliance. Nonetheless, the
available studies of compliance in the treatment of
PID consistently indicate that compliance is poor,
with substantial proportions of patients failing to
18 INFECTIOUS DISEASES IN OBSTETRICS AND GYNECOLOGY
DOXYCYCLINE OR OFLOXACIN FOR OUTPATIENT PID? ROSENBERG AND WAUGH
0 0 0 0 0 0 0 0 0
EE EEE
complete their medication and side effects increas-
ing noncompliance. 10,11
Second, the relationship between cure and vary-
ing levels of compliance is unknown. We consid-
ered 3 possibilities to address this uncertainty. For
example, such a relationship may be linear (as-
sumption A, Fig. 1) or "S"-shaped (assumption B,
Fig. 1), with an initially low cure rate that increases
rapidly over a short period and levels off after a
certain threshold of compliance has been attained.
Or, cure may be achieved rapidly with a relatively
low level of compliance (the logistic curve, as-
sumption C, Fig. 1). However, other relationships
may exist. In addition, the precise relationship may
vary according to individuals. Nevertheless, our
finding that only minimal increases in compliance
are necessary to markedly reduce the costs associ-
ated with PID sequelae, regardless of the relation-
ships examined, suggests that patient compliance
whether an antibiotic is used as prescribed
considerably overshadows the questions of the
change in probability of cure at a specific period of
time.
The baseline compliance rate of 50% compliance
with doxycycline may be lower than that encoun-
tered in some groups. Although the study from
which this figure was taken is the only one we were
able to locate that directly addressed compliance for
PID diagnoses, it involved patients at an urban
emergency room. It is, however, similar to the
63% compliance rate noted for erythromycin in
PID treatment from a study conducted in an STD
clinic. In addition, both studies involved a 7-day
regimen, so the compliance rates probably overesti-
mate the level of compliance expected for the 14-
day regimen recommended by the CDC. Even if
compliance differs from the baseline that we used,
our sensitivity analysis confirmed our basic conclu-
sions regarding the importance of compliance and
savings attributable to a drug with improved com-
pliance.
Compliance is obviated as an issue with azithro-
mycin, which is effective against chlamydial cervi-
citis as an orally administered, single dose. How-
ever, insufficient clinical experience precludes our
judging its efficacy in PID, as reflected by the fact
that this drug is not yet included as one of the
CDC's recommended treatments for PID.
The likelihood of a woman's coming to medical
attention depends in part on her symptoms. Since
INFECTIOUS DISEASES IN OBSTETRICS AND GYNECOLOGY 19
DOXYCYCLINE OR OFLOXACIN FOR OUTPATIENT PID? ROSENBERG AND WAUGH
infection with C. trachomatis is less likely than
Neisseria gonorrhoeae to cause symptoms, the fre-
quency of coinfection will affect whether a woman
is treated. If women are not treated, the issue of
compliance is moot. For an individual who receives
treatment, however, the probability of compliance
vs. the immediate cost of treatment and later
costs of sequela due to inadequate treatment is im-
portant.
In this analysis, we approached costs from a
broad perspective that included future as well as
present costs. Although some settings, notably
health departments, may focus on the immediate
issue of antibiotic cost, such a view is narrow sim-
ply because others may bear the costs of future
complications. From the broad perspective of a
health care system, including comprehensive health
providers such as HMOs, the argument for the use
of antibiotics that decrease long-term costs is co-
gent. Other means of improving compliance need
to be considered, including the physician's encour-
agement about the importance of the antibiotics,
written materials to reinforce this information, and
novel approaches to counseling, such as peer coun-
selors for adolescents. 1,
With more information on compliance with a
particular antibiotic regimen, the clinician may be
able to tailor treatment to each individual patient,
based in part on the patient's payment source for
prescriptions. Office-based physicians must judge
whether the patient, in the absence of insurance,
federal support, or some other copayment plan,
would be likely to have a prescription for ofloxacin
or any other more expensive treatment filled. Cli-
nicians working in managed care, publicly funded,
or other capitation care environments should con-
sider prescribing the alternative to doxycycline
should it in fact prove to increase levels of compli-
ance. By reducing the number of failed treatments
and PID sequelae, the clinician also reduces the
total costs that the health care system and potentially
his or her individual institution may later absorb.
ACKNOWLEDGMENTS
We are grateful for the suggestions of Peter Ma-
zonson, M.D., and anonymous reviewers. This
work was funded in part by a grant from Ortho-
McNeil Pharmaceutical.
REFERENCES
1. Washington AE, Katz P: Cost of and payment source for
pelvic inflammatory disease: Trends and projections, 1983
through 2000. JAMA 266:2565-2569, 1991.
2. Kiviat NB, Wolner-Hanssen P, Peterson M, et al.: Lo-
calization of Chlamydia trachomatis infection by direct
immunofluorescence and culture. Am J Obstet Gynecol
154:865-873, 1986.
3. Bowie WR, Jones H: Acute PID in outpatients: Associa-
tion with Chlamydia trachomatis and Neisseria gonor-
rhoeae. Ann Intern Med 95:685-688, 1981.
4. Moiler BR, Mardh PA, Ahrons S, Nussler E: Infection
with Chlamydia trachomatis, Mycoplasma hominis, and
Neisseria gonorrhoeae in patients with acute pelvic inflam-
matory disease. Sex Transm Dis 8:198-202, 1981.
5. Svensson L, Westrom L, Ripa KT, Mardh PA: Differ-
ences in some clinical and laboratory parameters in acute
salpingitis related to culture and serological findings. Am
J Obstet Gynecol 138:1017-1021, 1980.
6. Mardh PA, Ripa KT, Svensson L, Westron L: Chlamy-
dia trachomatis infection in patients with acute salpingitis.
N EnglJ Med 296:1377-1379, 1977.
7. Kahn JG, Walker CK, Washington AE, Landers DV,
Sweet RL: Diagnosing pelvic inflammatory disease.
JAMA 266:2594-2604, 1991.
8. Medical Economics Data, Inc.: Drug Topics Red Book
1993. Montvale, NJ: Medical Economics Data, Inc.,
1993.
9. Cramer JA, Mattson RH, Prevey ML, Scheyer RD,
Ouellette VL: How often is medication taken as pre-
scribed? A novel assessment technique. JAMA 261:3273-
3277, 1989.
10. Brookoff D, Kellerman A, Thorpe E: Compliance with
doxycycline in outpatient treatment of pelvic inflamma-
tory disease at an urban municipal emergency department
(abstract). Sex Transm Dis 2 I:S123, 1994.
11. Katz BP, Zwickl BW, Caine VA, Jones RB: Compliance
with antibiotic therapy for Chlamydia trachomatis and
Neisseria gonorrhoeae. Sex Transm Dis 19:351-354, 1992.
12. Martens MG, Gordon S, Yarborough DR, Faro S,
Binder D, Berkeley M: Multicenter randomized trial of
ofloxacin versus cefoxitin and doxycycline in outpatient
treatment of pelvic inflammatory disease. South Med J
86:604-610, 1993.
13. Wendel GD, Cox SM, Bawdon RE, Theriot SK, Heard
MC, Nobles BJ: A randomized trial of ofloxacin versus
cefoxitin and doxycycline in the outpatient treatment of
acute salpingitis. Am J Obstet Gynecol 164:1390-1396,
1991.
14. CDC: Pelvic inflammatory disease: Hospitalizations
among women 15-44 years of age, United States, 1980-
1991. National Hospital Discharge Survey. National Cen-
ter for Health Statistics, CDC, Washington, DC.
15. Westrom L: Incidence, prevalence, and trends of acute
pelvic inflammatory disease and its consequences in in-
dustrialized countries. Am J Obstet Gynecol 138:880-
892, 1980.
20 INFECTIOUS DISEASES IN OBSTETRICS AND GYNECOLOGY
DOXYCYCLINE OR OFLOXACIN FOR OUTPATIENT PID? ROSENBERG AND WAUGH
16. Dodson M: Antibiotic regimens for treating acute pelvic
inflammatory disease: An evaluation. J Reprod Med 39:
285-296, 1994.
17. Centers for Disease Control and Prevention. 1993 Sexu-
ally transmitted diseases treatment guidelines. MMWR
1993; 42(No. RR-14):75-83.
18. Jay MS, Durant RH, Shoffitt T, Linder CW, Litt IF:
Effect of peer counselors on adolescent compliance in use
of oral contraceptives. Pediatrics 73:126-131, 1984.
INFECTIOUS DISEASES IN OBSTETRICS AND GYNECOLOGY

				
